# Mountain wetland soil carbon stocks of Huascarán National Park, Peru

**DOI:** 10.3389/fpls.2023.1048609

**Published:** 2023-04-25

**Authors:** Rodney A. Chimner, Sigrid C. Resh, John A. Hribljan, Michael Battaglia, Laura Bourgeau-Chavez, Gillian Bowser, Erik A. Lilleskov

**Affiliations:** ^1^ Michigan Technological University, College of Forest Resources and Environmental Science, Houghton, MI, United States; ^2^ University of Nebraska Omaha, Department of Biology, Omaha, NE, United States; ^3^ Michigan Tech Research Institute, Michigan Technological University, Ann Arbor, MI, United States; ^4^ Department of Ecosystem Science and Sustainability, Colorado State University, Fort Collins, CO, United States; ^5^ United States Department of Agriculture (USDA) Forest Service, Northern Research Station, Climate, Fire and Carbon Cycle Sciences Unit, Houghton, MI, United States

**Keywords:** bofedales, puna, peatlands, tropics, wet meadows

## Abstract

Although wetlands contain a disproportionately high amount of earth’s total soil carbon, many regions are still poorly mapped and with unquantified carbon stocks. The tropical Andes contain a high concentration of wetlands consisting mostly of wet meadows and peatlands, yet their total organic carbon stocks are poorly quantified, as well as the carbon fraction that wet meadows store compared to peatlands. Therefore, our goal was to quantify how soil carbon stocks vary between wet meadows and peatlands for a previously mapped Andean region, Huascarán National Park, Peru. Our secondary goal was to test a rapid peat sampling protocol to facilitate field sampling in remote areas. We sampled soil to calculate carbon stocks of four wetland types: cushion peat, graminoid peat, cushion wet meadow, and graminoid wet meadow. Soil sampling was conducted by using a stratified randomized sampling scheme. Wet meadows were sampled to the mineral boundary using a gouge auger, and we used a combination of full peat cores and a rapid peat sampling procedure to estimate peat carbon stocks. In the lab, soils were processed for bulk density and carbon content, and total carbon stock of each core was calculated. We sampled 63 wet meadows and 42 peatlands. On a per hectare basis, carbon stocks varied strongly between peatlands (avg. 1092 MgC ha^-1^) and wet meadows (avg. 30 MgC ha^-1^). Overall, wetlands in Huascarán National Park contain 24.4 Tg of carbon with peatlands storing 97% of the total wetland carbon and wet meadows accounting for 3% of the wetland carbon in the park. In addition, our results show that rapid peat sampling can be an effective method for sampling carbon stocks in peatlands. These data are important for countries developing land use and climate change policies as well as providing a rapid assessment method for wetland carbon stock monitoring programs.

## Introduction

The ability of wetlands to store soil carbon is a well-established ecosystem function, driven primarily by waterlogging and the resulting inhibition of decomposition (Nahlik and Fennessy, 2016). As a result, wetlands contain a high proportion of the Earth’s total soil carbon relative to their surface area (Kolka et al., 2018). Wetlands also provide many additional ecosystem functions and values, such as improved water quality and storage, nutrient transformation and storage, habitat, and grazing. Most wetlands are found in low-lying areas, but they are also found in mountainous landscapes (Chimner et al., 2010; Cooper et al., 2012). However, only a small fraction of mountain wetlands are mapped, and even fewer have carbon stock estimates (Hribljan et al., 2016; Hribljan et al., 2017).

Wetlands are abundant in many mountain systems due to high precipitation from orographic uplift, abundant groundwater, and cooler temperatures compared to the surrounding lowlands (Cooper et al., 2012). Mountain wetlands often occur in discrete elevation zones due to differences in water availability, landforms suitable for wetland development, and watershed size (Chimner et al., 2010). Mountains also contain a variety of wetland types due to high spatial variability in topography and water availability, including marshes, wet meadows, peatlands, riparian systems, and vernal pools (Cooper et al., 2012). Recent mapping efforts show that the tropical Andes have a high abundance of wetlands compared to many other mountain ranges (Chimner et al., 2010; Hribljan et al., 2017; Chimner et al., 2019). For example, 18% of the area mapped in the Ecuadorian Andes was mapped as wetlands (Hribljan et al., 2017), and 10% of the area was mapped as wetlands in Huascarán National Park, Peru (Chimner et al., 2019).

Andean wetlands can be near monocultures of cushion plants, mixed cushion plants with smaller components of mosses, forbs, shrubs, and graminoids, or dominated by graminoids with few to no cushion plants present (Chimner et al., 2019). These cushion plants-members of the Juncaceae, Asteraceae, and Plantaginaceae families (Cooper et al., 2010; Salvador et al., 2014; Chimner et al., 2019)-are a globally unique wetland vegetation type. Cushion plants have low, dense growth forms that trap heat and increase canopy moisture by reducing wind shear and evapotranspiration (Billings and Mooney, 1968; Cavieres et al., 2007). Cushion plants also have long aerenchymatous roots that extend below the water table to capture deeper soil moisture and nutrients from saturated peat (e.g., Suárez et al., 2021).

There are two main types of wetlands in the tropical Andes, wet meadows and peatlands, which can occur individually or together in wetland complexes (Chimner et al., 2019). Wet meadows are mineral soil wetlands with seasonally saturated soils (Schook et al., 2020) dominated by herbaceous plants (Cooper et al., 2012). They are common in the Sierra Nevada (Loheide et al., 2009) and Rocky Mountains (Cooper et al., 2012) of the USA, and in the central Andes (Chimner et al., 2020). It can be very difficult to visually distinguish peatlands from wet meadows in the Andes because of the similarity of the plant communities that grow on both, which can be cushion or graminoid dominated, and the fact that they can occur together in large complexes (Chimner et al., 2019). Our mapping showed that approximately half of all wetlands in Huascarán National Park, Peru, are wet meadows and the other half are peatlands. However, it is unknown at the landscape scale how much carbon wet meadows store compared to peatlands in the Andes.

Globally, peatlands, a class of wetlands defined as having more than 40 cm of organic soil, have the largest soil carbon stocks of any wetland type, and perhaps the largest of any ecosystem (Page and Baird, 2016). Current estimates suggest that peatlands store about one-third of the world’s soil carbon in about 4% of the land area (UNEP, 2022). Most peatlands are located in the boreal region, with much of the remainder in tropical lowland peatlands (Page et al., 2011). Andean alpine peatlands are common throughout the South American tropics from 3,000 to >5,000 masl (Cooper et al., 2010; Hribljan et al., 2016). They range in age from 1,000 to 10,000 years, with most having originated between 3,000 and 5,000 years ago (Earle et al., 2003; Chimner and Karberg, 2008); have very thick peat deposits averaging ~5-6 m; and have high C/area densities, with several sampled peatlands having more than 10 m of peat.

As more regions of the world, especially the tropics, are sampled for peatland carbon stocks to inform land use and climate change policies and to improve carbon stock monitoring programs, new methods are needed to enable faster soil sampling and carbon stock calculation in an efficient and cost-effective manner. Currently, sampling the entire peat column and quantifying peat in many finely divided subsamples along the length of the column is the most accurate method because the entire peat core is collected and processed to quantify total peat carbon stocks. However, this process is time-consuming, logistically challenging, and expensive, especially in landscapes with dense peat accumulations and in remote areas (Chimner et al., 2014). One rapid sampling method calculates peat carbon stocks by sampling only the thickness of the peat and multiplying it by a regionally appropriate carbon density value (Chimner et al., 2014). This method is fast, requires no sampling or laboratory analysis, and has been shown to be 85-90% accurate compared to sampling the entire peat profile. However, this method has never been fully tested in a field sampling campaign. Our main research objective was to quantify soil carbon stocks for two of the most important mountain wetland types in the Andes, wet meadows and peatlands, because it is unknown at the landscape scale how much carbon wet meadows store compared to peatlands in the Andes. We hypothesized that although peatlands and wet meadows have roughly equal area coverage in our study area, peatlands will store the vast majority of soil carbon on a landscape scale. Our secondary objective was to test our rapid peat sampling protocol to facilitate sampling in remote areas.

## Materials and methods

### Study area

**Figure 1:**
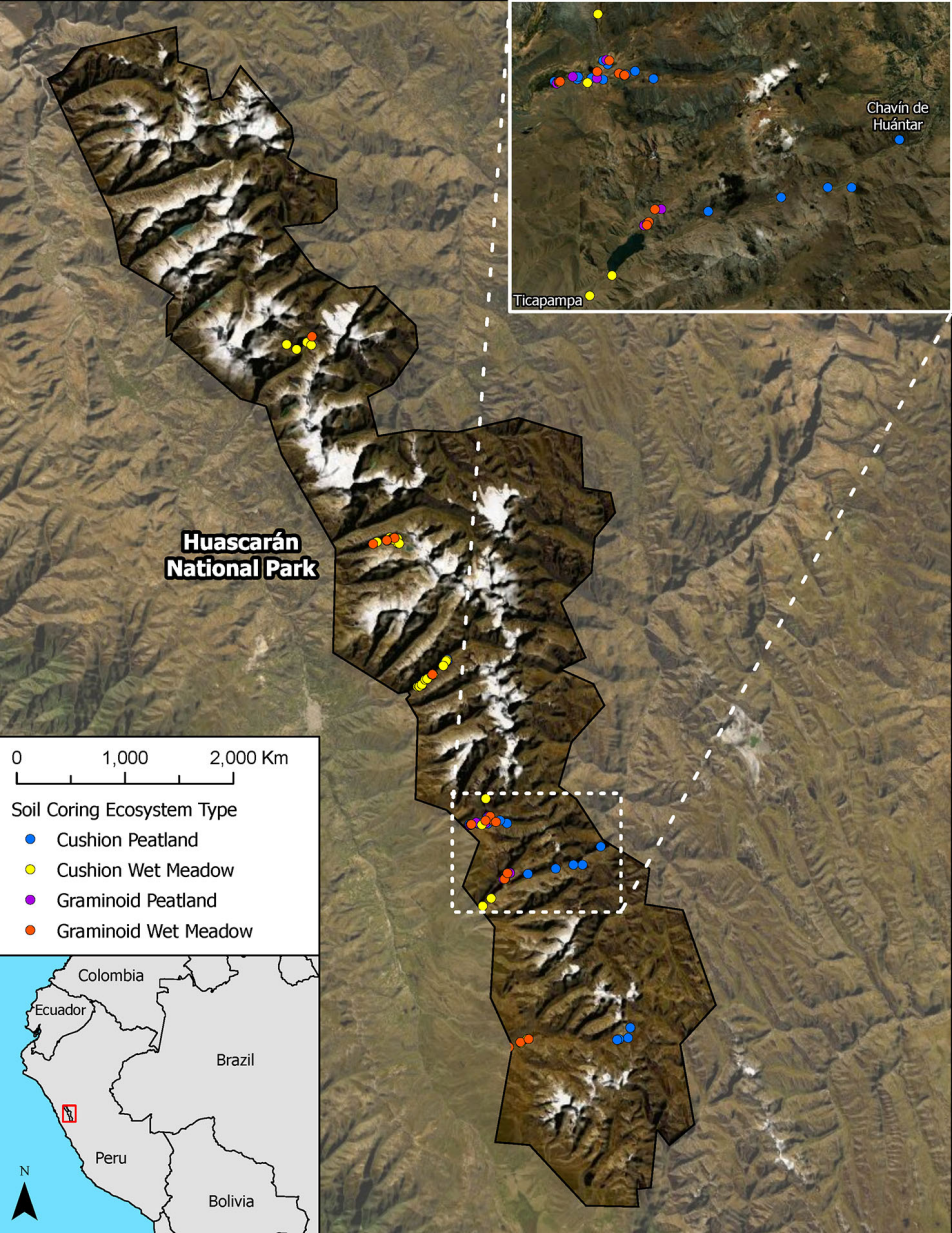
Location map Huascarán National Park, Peru showing sampling locations.

This study was conducted in Huascarán National Park, in the department of Ancash in the north-central Andes of Peru (**Figure 1**). The park is located in the Cordillera Blanca and has a core area of 340,000 ha, with peaks ranging from 5,000 to 6,768 masl. The Cordillera Blanca contains approximately 755 glaciers and 830 lakes of glacial origin.

In a previous study, we mapped wetlands throughout the park using multi-date, multi-sensor radar and optical imagery (Landsat TM/PALSAR/RADARSAT-1) and topographic data (SRTM DEM-TPI) combined with field validation (see Chimner et al., 2019 for details). Wetlands were grouped into two major types, peatlands and wet meadows, with two vegetation types for each group for a total of four wetland types: 1) cushion plant peatlands, 2) graminoid peatlands, 3) cushion plant wet meadows, and 4) graminoid wet meadows. The total wetland area mapped in Huascarán NP was 31,846 ha, with cushion plant peatlands being the most abundant wetland type occupying 6.3% of the park, followed by graminoid wet meadows (3.5%) and cushion plant wet meadows (1.3%) and graminoid peatlands (<1%).

### Field data collection

**Figure 2:**
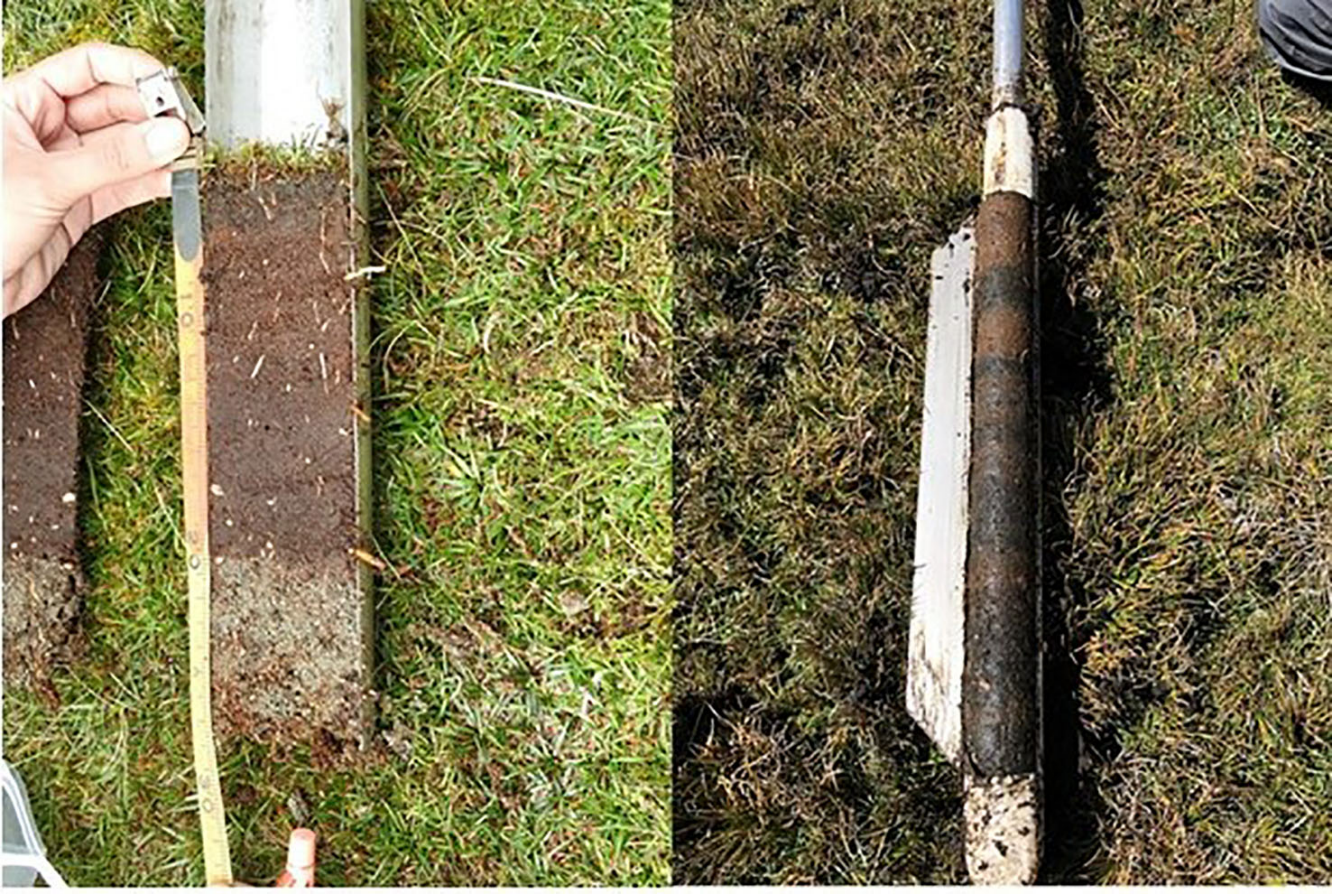
Image showing the difference in soil carbon between wet meadows (left) and peatlands (right).

We collected soil samples to calculate carbon stocks in the wetlands of Huascarán NP using a stratified random sampling scheme. Because the park is large and many areas are very difficult to access (Brus et al., 2011; Clifford et al., 2014), we identified six large, accessible regions within the park as representative sampling areas. Random points were selected within previously mapped wetlands that were also within 3 km of a road or accessible by trails. We sampled points for each of four wetland types: cushion peat, graminoid peat, cushion wet meadow, and graminoid wet meadow within each region (see Chimner et al., 2019). Random points were selected from the resulting polygons using the Random Points in Polygons tool in QGIS software (version 3.6). A total of 250 random points were generated for each class. Seventy-five points each were generated for cushion peatlands, cushion wet meadows, and graminoid wet meadows. Graminoid wetlands were rare on the landscape (<1% of the total area), so only 25 random points were generated for this class. In the field, we sampled soils at as many of the random points as time permitted during our sampling campaign. Wet meadows were sampled using a 3.175 cm x 101.6 cm gouge auger (AMS, American Falls, ID, USA), which was inserted into the soil until the subsoil was reached by direct sampling (**Figure 2**). Once collected, soils were cut into 10 cm sections in the field, placed in labeled Whirl-Pak bags, and stored in a refrigerator.

We used a rapid peat sampling method to estimate peat carbon stocks (the “thickness-only method” of Chimner et al., 2014). In this method, the thickness of the peat profile is measured and multiplied by a peat carbon density factor to calculate the total carbon mass (Chimner et al., 2014). We used a peat carbon density factor of 0.0362 gC cm-3 derived from 26 complete peat cores sampled throughout the northern Andes, including 6 previously collected in Huascarán NP (Hribljan et al., in review). The thickness-only method is very rapid and does not require soil sampling or laboratory analysis. Thickness was measured by inserting a metal probe into the peat soil until it contacted the underlying mineral soil. In a previous study, this method was found to be 85-90% accurate compared to collecting the entire peat profile (Chimner et al., 2014). We chose this rapid peat sampling method because of the length of time it takes to core peat in this region. For example, we collected 6 peat cores from Huascaran NP (Hribljan et al., in review) and it took over 4 hours to collect a core. In addition, it was logistically challenging to ship the peat to the laboratory in the United States for processing. The rapid sampling technique required only 10-15 minutes per sample and allowed us to estimate carbon content with much higher repeatability than coring alone.

We cored four additional peatlands to directly assess the accuracy of the rapid sampling method on the same cores. This method involved using a Russian peat corer (Aquatic Research Instruments, Hope, Idaho, USA) and sampling peat in 50 cm increments until mineral material was reached (Hribljan et al. in review). Peat cores were stored in Whirl-Pak bags and placed in a refrigerator. At the end of sampling, all soils were transported by air in a cooler to the Michigan Tech Wetland Ecology and Restoration Lab, where they were immediately frozen (-23°C) until analysis.

### Laboratory analysis

In the laboratory, soils were cut into 5 cm increments and dried in a convection oven at 65°C until a constant mass was achieved. Dry bulk density (g cm-3) was calculated by dividing the oven dried soil mass by the original sample volume. Soil organic matter content was determined for all core sections by loss on ignition (LOI) at 550°C for 5 h (Chambers et al., 2011). LOI was converted to %C using the following equation (LOI*0.52 = %C) as calculated for Andean peat (Hribljan, in review). Carbon mass was then calculated for each soil section by multiplying bulk density * 5 cm depth interval * %C. The total carbon stock of each peat core was calculated by summing the carbon mass of each 5 cm soil section along the length of the entire soil core.

Total carbon stocks for Huascarán NP were calculated for each wetland type by multiplying the mean carbon stock per unit area for each wetland type by the previously mapped wetland area (Chimner et al., 2019). Wetland area was mapped using multi-date optical data from Landsat and radar imagery from ALOS PALSAR and Radarsat-1, and was used in combination with digital elevation model (DEM) data in map classification (see Chimner et al., 2019 for details).

### Statistics

An analysis of variance (ANOVA) and t-test were conducted using SYSTAT (SYSTAT Software, San Jose, CA) to test for differences in soil carbon parameters. A stratified random sampling design was used with wetland types as the main plot factor. We also used linear regression in Sigmaplot 13 (SYSTAT Software, San Jose, CA) to determine the relationships between carbon stock estimates of peat coring and probing.

## Results

We sampled 63 wet meadows and 42 peatlands in the study regions (**Figure 2**). The organic horizon thickness of wet meadows averaged 22 cm (2 cm - 120 cm), with cushion wet meadows being ~8 cm thicker on average than graminoid wet meadows (**Table 1**). Using all 42 peatlands sampled, the mean peat thickness was 280 cm (78 cm - 1068 cm), with organic horizons of cushion peatlands being ~50 cm thicker than graminoid peatlands (**Table 1**). Bulk density did not vary significantly between wetland types, but %C did, with peatlands having higher %C compared to wet meadows and cushion peatlands having higher %C than graminoid wet meadows (**Table 1**).

**Table 1:** Wetland soil properties, area (Chimner et al., 2019), and total carbon stocks (standard errors in parenthesis).

Wetland Type	Thickness (cm)	Carbon (g/kg)	Bulk Density (Mg/m^3^)	C-Stock (MgCha)	Wetland Area (ha)	C-Stock (Tg)
Peatland- Cushion (32)	294.8^A^ (35.0)	29.1^A^ (1.5)	0.17^A^ (0.02)	1099.3^A^ (149.0)	14,917	16.4 (8.8)
Peatland –Graminoid (10)	233.0^A^ (38.2)	26.8^AB^ (n/a)	0.10 ^A^ (n/a)	787.1^A^ (116.3)	338	0.16 (0.08)
Wet Meadow –Cushion (42)	24.7^B^ (3.2)	18.7^B^ (1.6)	0.14 ^A^ (0.01)	39.9^B^ (5.0)	4,339	0.17 (0.05)
Wet Meadow- Graminoid (21)	16.6^B^ (2.0)	12.2^C^ (1.3)	0.18 ^A^ (0.08)	26.7^B^ (3.9)	12,253	0.33 (0.25)

Total mapped wetland area = 31,846 ha (peatlands=48%, wet meadows=52%). Thickness indicates the depth of organic horizons. Letter superscripts denote significant differences (P < 0.05) between wetland types.

**Figure 3:**
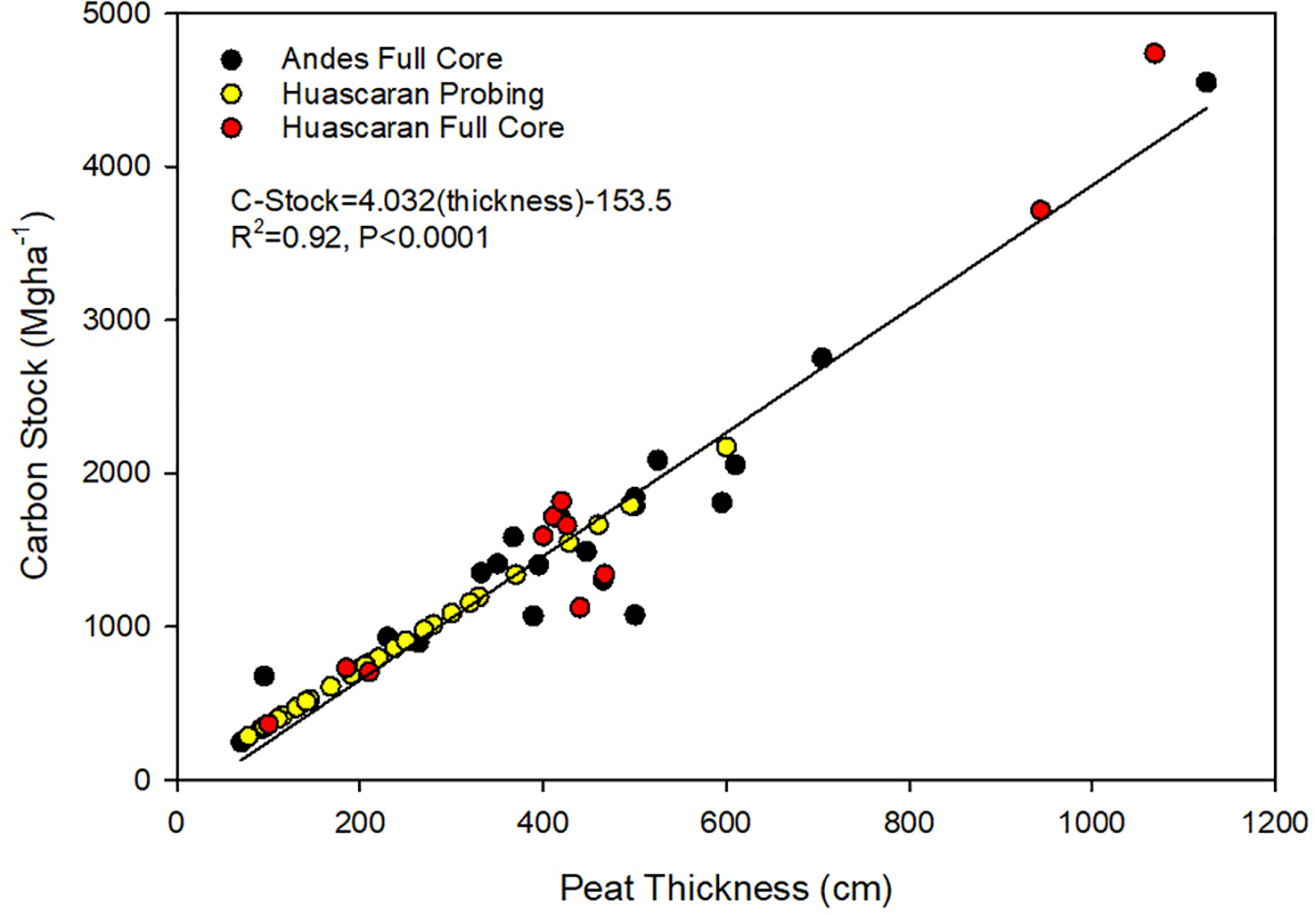
Correlation between full peat cores from across the Andes (black circles: Hribljan et al., 2023), full cores from this study (red circles), and probe values from this study (yellow circles).

The four peat cores used for the peatland carbon stock method comparison averaged 1,199 MgC ha-1 and were not significantly different from the probing only method, which had an average of 1,223 MgC ha-1 for the same cores using the thickness*C factor (a 2% difference: t-test p-value = 0.96). In addition to the peat cores, we also examined 32 additional peatlands (9 graminoid and 23 cushion peatlands) (**Figure 3**). There was a linear correlation between peat thickness and carbon stocks, with the probing only sites showing a good correlation (R2 = 0.92, p-value < 0.0001) in total C compared to full cores from collections both in Huascarán NP and across the tropical Andes (**Figure 3**).

**Figure 4:**
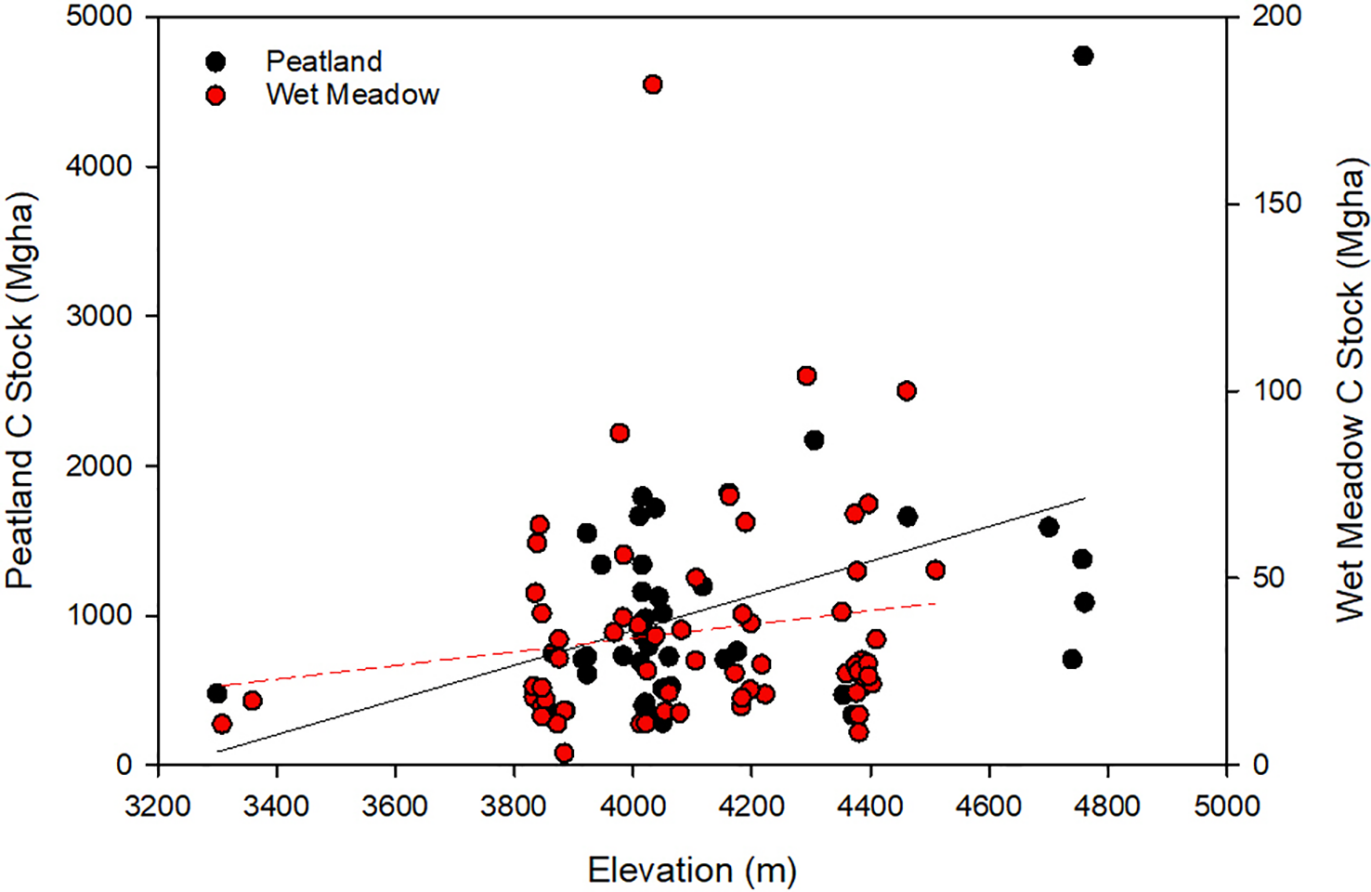
Correlation between peatland carbon stocks (Black circles: Peatland C-Stock=1.16(elevation)-3736.3, R2 = 0.19, P<0.004) and wet meadows (Red circles: Wet meadow C-Stock=0.018(elevation)-39.3, R2 = 0.02, P<0.22) and elevation. Note the difference in scale between the peat samples (left y axis) and the wet meadow samples (right y axis).

On a per hectare basis, carbon stocks differed greatly between peatlands and wet meadows. Peatland carbon stocks ranged from 278 to 4,740 MgC ha-1 with a mean of 1092 MgC ha-1, while wet meadows ranged from 3 to 181 MgC ha-1 with a mean of 30 MgC ha-1 (**Table 1**). There was no significant difference between peatland and wet meadow types. Both peatlands and wet meadows showed a general trend of increasing carbon stocks with increasing altitude (**Figure 4**).

We calculated total park-wide carbon stocks by multiplying the mapped area for each wetland type (Chimner et al., 2019) by our average calculation of wetland-specific carbon stocks (**Table 1**). Overall, wetlands in Huascarán NP store 24 Tg of carbon (**Table 1**). Peatlands store 97% of the total belowground carbon stock of wetlands in the park, with cushion plant peatlands accounting for 95% of wetland carbon storage (**Table 1**). Graminoid peatlands, graminoid wet meadows and cushion plant wet meadows each store ~1% of the total wetland soil carbon in the park (**Table 1**).

## Discussion

### Carbon stocks of Andean mountain wetlands

Mountain peatlands, like all peatlands, can be large carbon sinks. Our mean peat depth throughout Huascarán NP is 2.8 m with a mean C stock of 1,092 Mg ha-1. These values are similar to our sampling in the Ecuadorian Paramo, where the mean peat depth was 3.8 m with a mean C-stock of 1,282 Mg ha-1 (Hribljan et al., 2016). Despite the fact that Andean peatlands occur at high elevations and under perennially cold conditions, they have similar C stocks to peatlands in the lowland Peruvian Amazon, which average 1,037 Mg ha-1 in the Pastaza Marañón foreland basin, the largest known peatland complex in the Amazon basin (Draper et al., 2014; Bourgeau-Chavez et al., 2021). Andean peatlands are deeper than temperate mountain peatlands, with average depths of 1.5 m (Chimner et al., 2010) and ~1 m (Wolf and Cooper, 2015) in the Colorado Rockies and Sierra Mountains, respectively.

The tropical Andes are divided into northern (páramo) and central (puna) bioregions (Cleef, 1979). The páramo ecoregion of Colombia, Venezuela, Ecuador, and northern Peru is located in the humid equatorial Andes and is characterized by cool and wet conditions with no distinct dry season (Luteyn, 1999). In contrast, the puna ecoregion of central Peru, Bolivia, and northern Chile and Argentina (Young et al., 1997; Earle et al., 2003) is characterized by stronger seasonality with distinct wet and dry seasons. Although our sampling in the two regions, the Puna and the Páramo, shows that peat depth and C stocks are similar, total peatland C stocks at the landscape level may not be equivalent in the two regions. Mapping work in the páramo of north-central Ecuador using multi-date, multi-sensor remote sensing techniques found that all sampled wetlands were peatlands (Hribljan et al., 2017), while in the puna of Huascarán NP, 57% of mapped wetlands were peatlands (Chimner et al., 2019). In parallel, the total C stocks of peatlands in these two regions differ: Huascarán NP has a total C stock of 17 Tg (1,092 Mg ha-1), while the mapped Ecuadorian area contained 128 Tg (2,123 Mg ha-1) (Hribljan et al., 2017). However, these two studies only examined two small regions in the puna and páramo, which may not be representative of the regions as a whole. For example, Huascarán NP is part of the Cordillera Blanca, which has high peaks ranging from 5,000 to 6,768 masl (including Peru’s highest peak, Huascarán Sur) and 755 glaciers, representing 25% of all tropical glaciers (Mark et al., 2010). Thus, more than half of the mapped area was either rock or glacier, minimizing the area available for peatland formation. This rock or glacier area is much larger than in Ecuador, where only 10% of the landscape was mapped as rock or glacier. The true differences between puna and páramo should become clear when national peatland maps are completed, which are currently underway for Colombia, Ecuador, and Peru.

Although all wetlands store large amounts of carbon, some wetland types store more than others. For example, in North America, mineral soil wetlands contain ~20% of total wetland carbon, while organic soil peatlands store ~80%, even though mineral soil wetlands make up 40% of wetland area (Kolka et al., 2018). In the present study, we found an even greater disparity, with wetland C stocks of 0.5 Tg compared to 17 Tg for peatlands. Across Huascarán NP, wet meadows represented 52% of the wetland area but stored only 2% of the carbon, while peatlands represented 48% of the wetland area but stored 98% of the carbon. This suggests that ongoing efforts to quantify Andean wetland carbon stocks should focus on peatlands.

However, the fact that wet meadows do not store as much soil carbon as peatlands does not diminish their importance. Wet meadows are important because they provide many other benefits, including high quality habitat, nutrient sinks and transformations, water storage and cycling, and grazing areas (Chimner et al., 2020). Wet meadows are often used for grazing because they are seasonally wet and have stable soils compared to peatlands.

### Rapid peat sampling

Rapid peat sampling can be a useful tool when trying to sample large areas for calculating peatland carbon stocks (Chimner et al., 2014). An early example of rapid peat sampling was an intermittent method developed for sampling peatlands in Indonesia because many tropical peatlands are very deep and it was logistically difficult to collect, transport, and analyze all the peat. The method subsamples 5 cm sections of peat in the field from depths of 5-10, 20- 25, 37.5-42.5, 72.5-77.5, 197.5-202.5 cm, and then every 3 m for the remaining core (e.g., 497.5-502.5, 797.5-802.5, etc.) (Kauffman and Donato, 2012). In an evaluation of six rapid peat sampling methods, Chimner et al. (2014) found that several rapid peat sampling techniques were >85% accurate compared to whole core harvesting. Chimner et al. (2014) also found that accuracy was high for sampling numerous peatlands, but could be inaccurate for a single peatland, suggesting that rapid peat sampling is best suited for large peat inventories.

For this study, we chose to use one of the rapid peat sampling protocols developed in Chimner et al. (2014), the peat probing only method. We found that of the four cores that we probed and collected a full core, probing was ~90% accurate compared to collecting the full cores. The main advantage of rapid sampling is that peat does not need to be collected, transported, and analyzed in a lab, which results in a significant cost and time savings, and allows for more sampling across the sample area, which can result in a more accurate carbon stock estimate. For example, our estimate of carbon stocks in the Ecuadorian Paramo (Hribljan et al., 2017) was done with only 10 peat cores, while this paper also used 10 complete peat cores (4 collected in this study and 6 collected previously), but we were able to supplement with an additional 32 rapid sampling points spread across the park, greatly increasing the spatial resolution.

Although there are many advantages to using a rapid peat sampling method, errors can occur if incorrect peat thickness measurements or an inaccurate carbon density factor are used. We used a peat carbon density factor (0.0362 gC cm-3) derived from 26 complete peat cores sampled throughout the northern Andes (Hribljan et al., in revision). It was important to develop a new carbon density factor because this factor is lower than peatlands in North America (Chimner et al., 2014; Magnan et al., 2023), likely due to the greater amount of mineral soil and ash entering these peatlands (Hribljan et al., in revision). The other aspect unique to Andean peatlands is the dominance of cushion plants (Cooper et al., 2010), which have a dense, compact growth form, which also contributes to the need for a new density factor for Andean peatlands (Billings and Mooney, 1968).

Probing to determine the correct depth of peat can also be a source of error. The main problem we found is that it is difficult to probe deeper than 5-6 m. Andean peats are denser than many other peats due to volcanic ash and high mineral content from sediment deposition from steep mountain slopes adjacent to the peatlands. Another difficulty that can be encountered is that it can be difficult to detect the boundary between the bottom of the peat layer and the underlying mineral sediment with a peat probe, especially if there are mineral layers interbedded in the peat (e.g., Chimner and Karberg, 2008). It is easier to sample thickness in peatlands that 1) do not have thick layers of interbedded minerals, and 2) are located directly over sandy or loamy material - the sand “crunches” and rejects the probe. In addition, similar to peat coring, peat probing can introduce errors due to the uneven thickness of the peat (Parsekian et al., 2012; Comas et al., 2017; Beucher et al., 2020). For example, if peat probing always occurs in the deepest part of the peat, then C stocks will be overestimated. Therefore, representative samples or multiple samples per site could be taken from each peatland to minimize this error.

The rapid peat sampling protocol used in this study was developed for field forestry inventory crews such as the USDA Forest Service Forest Inventory and Analysis Program (FIA). Similar inventory crews in many countries collect field data on forest structure and biomass in peatlands, but typically do not collect data that can be used to estimate soil carbon stocks. However, by incorporating this rapid and simple peat probing technique, these crews can collect important peat stock data when plots occur in peatlands. However, a complication with this method is scaling up from point measurements to a peatland or region. In this study, we used a randomized sampling design to scale up to the park. However, there are other recent advances in airborne geophysical methods that are now being used to calculate peat thickness and carbon stocks in peatlands (e.g., Silvestri et al., 2019; Boaga et al., 2020). These methods have advantages over this rapid peat sampling protocol in that they can estimate peat thickness over large areas and account for variation in peat thickness across a site (Silvestri et al., 2019). This shows great promise for calculating peat C stocks in large peatland areas, such as Indonesia and the Amazon Basin. However, it is unclear how well these new techniques will work in the Andes, where there are tens of thousands of small peatlands scattered across complex mountain terrain.

In conclusion, our results suggest that ongoing efforts to map wetland carbon stocks in the Andes should focus more on peatlands and less on other wetland types, since peatlands store the vast majority of carbon. However, calculating carbon stocks in peatlands is difficult in the Andes due to the deep peat and very remote and rugged environments, requiring an efficient and accurate technique to develop peatland carbon stock estimates.

## Data availability statement

The raw data supporting the conclusions of this article will be made available by the authors, without undue reservation.

## Author contributions

RC developed the project, MB, JH, LB-C, and EL helped with sampling design, RC, SR and GB conducted the data collection, and RC did most of the analysis and writing with editing help by all. All authors contributed to the article and approved the submitted version. 
